# Automatic approach-avoidance tendencies as a candidate intermediate phenotype for depression: Associations with childhood trauma and the 5-HTTLPR transporter polymorphism

**DOI:** 10.1371/journal.pone.0193787

**Published:** 2018-03-16

**Authors:** Pascal Fleurkens, Agnes van Minnen, Eni S. Becker, Iris van Oostrom, Anne Speckens, Mike Rinck, Janna N. Vrijsen

**Affiliations:** 1 Behavioural Science Institute, Radboud University Nijmegen, Nijmegen, The Netherlands; 2 Psychotrauma Expertise Centrum (PSYTREC), Bilthoven, The Netherlands; 3 Department of Psychiatry, Radboud University Medical Centre, Nijmegen, The Netherlands; 4 Pro Persona: Institution for Integrated Mental Health Care, Nijmegen, The Netherlands; Radboud University Medical Centre, NETHERLANDS

## Abstract

Depression risk genes in combination with childhood events have been associated with biased processing as an intermediate phenotype for depression. The aim of the present conceptual replication study was to investigate the role of biased automatic approach-avoidance tendencies as a candidate intermediate phenotype for depression, in the context of genes (5-HTTLPR polymorphism) and childhood trauma. A naturalistic remitted depressed patients sample (N = 209) performed an Approach-Avoidance Task (AAT) with facial expressions (angry, sad, happy and neutral). Childhood trauma was assessed with a questionnaire. Genotype groups were created based on allele frequency: L_a_L_a_ versus S/L_g_-carriers. The latter is associated with depression risk. We found that remitted S/L_g_-carriers who experienced childhood trauma automatically avoided sad facial expressions relatively more than L_a_L_a_ homozygotes with childhood trauma. Remitted L_a_L_a_-carriers who had *not* experienced childhood trauma, avoided sad faces relatively more than L_a_L_a_ homozygotes with childhood trauma. We did not find a main effect of childhood trauma, nor differential avoidance of any of the other facial expressions. Although tentative, the results suggest that automatic approach-avoidance tendencies for disorder-congruent materials may be a fitting intermediate phenotype for depression. The specific pattern of tendencies, and the relation to depression, may depend on the genetic risk profile and childhood trauma, but replication is needed before firm conclusions can be drawn.

## Introduction

Major depressive disorder is highly prevalent and is associated with high personal and societal costs [1–3]. Recurrence of the disorder is very common [4]. It is therefore important to study underlying biological and cognitive risk factors for depression, in order to increase understanding of the etiology of depression and possible targets for intervention.

The serotonin transporter gene (SLC6A4), and especially a polymorphism in the promoter region (5-HTTLPR), have been linked to depressive symptoms [5]. The 5-HTTLPR polymorphism has a ‘short’ (S) and a ‘long’ (L) form, referring to the number of base pairs in the region. The function of the L allele is influenced by a single-nucleoid polymorphism or SNP (rs25531), which consists of an A and a G allele (L_a_ and L_g_). Importantly, the ‘short’ (S) form as well as the L_g_ variant have been associated with depression risk, because they restrict the transcriptional activity of the promoter, resulting in low functional expression of SLC6A4, and hence reduced re-uptake of serotonin [6].

Genetic expression probably depends on lifetime experiences [7]. Childhood trauma is a strong contributor to the development of psychopathology such as depression [8]. A recent meta-analysis [9] again confirmed life stressors to be a strong risk factor for depression. In contrast, the meta-analysis failed to show a main effect of 5-HTTLPR or an interaction of 5-HTTLPR x Stress on depression. The challenged validity of this stress sensitivity hypothesis by inconsistent results might stem from a restricted focus on depression phenotypes as the outcome of interest, as opposed to intermediate or transdiagnostic conditions [10,11]. Intermediate phenotypes are processes that are more proximal to the disorder than genes [7] and are thought to lay between the gene and clinically observable mental disorders. Studies into intermediate phenotypes are therefore considered a strong and reliable way of studying genetic influences and interactions with environmental factors [7,11,12].

Depression is characterized by biased processing of emotional information [13]. Importantly, genetic risk alleles and childhood trauma have been associated with biased processing as an intermediate phenotype for depression [11,14,15]. Most intermediate phenotype studies were conducted in healthy / community samples [10,16,17,18,19], and only some focused on clinical samples [20,21,22]. These studies typically point out that risk-allele carriers who experienced childhood trauma show most negative and least positive biased information processing. Cognitive processes such as attention, interpretation and memory have received most attention in this field. However, biased and aberrant behavioral approach-avoidance tendencies are also characteristic of and a risk factor for depression [23]. Specifically, avoidance is thought to limit the access to both negative and positive reinforcers, and promote negative information processing in depression. This seems especially relevant in the context of facial expressions, which communicate emotional states and direct behavior [24].

It is hypothesized that 5-HTTLPR is linked to these approach-avoidance tendencies. There is evidence that variation in 5-HTTLPR functioning is related to stress-sensitivity and response to negative cues. Beevers et al. [25], for example, found less attentional focus on negative compared to positive information in S/Lg-carriers, possibly indicating avoidance of negativity. Neurocognitive studies, furthermore, indicated that in response to negative information carriers of the short allele compared to carriers of the long allele 1) displayed a clear lateral shift of dorsolateral frontal activity to the right [26], and 2) showed heightened amygdale activity [27]. These neurocognitive responses are associated with avoidance-oriented motivation [26], indicating a mechanistic association between serotonin regulation and avoidance behavior. In line, recently, in a sample of PTDS patients, variation in the 5-HTTLPR polymorphism has been linked to avoidance behavior [28]. Corroborating evidence comes from pharmacological studies, where serotonergic medication has been found to reduce avoidance behavior in PTSD-patients [29].

Most studies on approach-avoidance behavior are based on self-report measures. A disadvantage of self-report is that it is limited to conscious processes, while missing out on more implicit processes [30,31] The Approach-Avoidance Task (AAT) [32] is a frequently used task to assess automatic approach-avoidance tendencies. Using the AAT, automatic approach-avoidance tendencies have been assessed in various disorders (e.g. Posttraumatic Stress Disorder (PTSD) [33]; fear of spiders: Klein, Becker, & Rinck [34]; social anxiety [35]; pathological skin picking [36]; and addictions [37], for a recent meta-analysis, see [38]). Although AAT results in depression are limited and inconsistent, automatic approach-avoidance tendencies are probably important in depression [39,40,41,42]. Seidel and colleagues [42], for example, found that depressed patients showed automatic avoidance in response to angry faces. Bartoszek and Winer [39] found that, in a student sample, those with depressive symptoms showed less automatic approach for positive pictures (non-faces) compared to neutral stimuli. This calls for further investigation.

In line with earlier intermediate phenotype findings, in the current conceptual replication we examined the hypothesized association of automatic approach-avoidance tendencies as a candidate intermediate phenotype for depression with the 5-HTTLPR polymorphism in interaction with childhood trauma. To our knowledge, the current study is the first study with this objective, and we therefore stress the tentative nature of the results and subsequent conclusions. Different from most other studies focusing on community samples, a naturalistic sample of remitted depressed patients was selected for the current study. This sample allows for the study of small genetic effects on intermediate phenotypes without the strong influence of current depressive symptoms [43,44]. In line with earlier studies, a sad mood induction procedure was used to activate depressotypic automatic processing and to align mood state [45,46].

Given the possible role of automatic approach-avoidance tendencies in depression, we explored automatic approach-avoidance tendencies as an intermediate phenotype for depression. Although we do not have previous genetic research to base our hypotheses on, based on clinical experiences, information processing theories in depression and the research by Seidel and colleagues [42], we expected avoidance of negative (sad, angry) facial expressions in remitted depressed S/L_g_-carriers who have experienced childhood trauma, as this represents the most vulnerable subgroup. Following Culverhouse and colleagues [9], we expected a main effect of childhood trauma on automatic approach-avoidance tendencies in depression.

## Materials and methods

### Participants

This study is part of the ‘Info in Genes’ study, which involves a total of 337 remitted depressed patients. In the ‘Info in Genes’ study, the role of cognitive biases and genetic susceptibility to depression is investigated by assessing biased processing in several domains in remitted depressed patients [20,21,22]. Included were those who met DSM-IV-TR criteria [1] for at least one previous Major Depressive episode (primary diagnosis). All remitted depressed individuals were recruited via the Department of Psychiatry of the Radboud University Medical Center. The following exclusion criteria were used: Current depressive episode, current or lifetime bipolar disorder and/or schizophrenia, current psychotic symptoms, substance abuse or dependence over the past 6 months, neurological disorder, sensorimotor handicap, intellectual disability, and deafness. Eligible participants were interviewed using the Structured Clinical Interview for the DSM-IV Axis-I disorders (SCID-I) [47,48] by trained professionals under the supervision of a psychiatrist (author AS). The SCID-I has fair test-retest reliability and fair-to-excellent inter-rater reliability [49]. AAT data were collected and available for 224 remitted depressed patients. The data of 3 patients with > 24% errors were removed from the dataset. Blood samples of eight patients were not available and genotyping failed for two participants. Another two participants were excluded because of a previous cardio-vascular accident and a recent transient ischaemic attack, resulting in a sample of 209 remitted depressed patients for data-analyses. The study was approved by the Dutch central medical ethics review board (P04.0599C), and all participants provided written informed consent.

### Measurements and materials

#### Mood induction

Before the AAT, all participants saw a highly emotional sad 12-minute film segment of the movie ‘Sophie’s Choice’ [50]. They were instructed to let the emotionality of the film influence their mood as much as possible and to maintain the sad mood state.

#### Approach-Avoidance Task

Color photos of 10 individuals (5 female, 5 male) from the Radboud Faces Database [51] were used. For each individual, one photo depicting an angry, sad, happy, and neutral expression each was selected. Additionally, 10 control pictures displaying a chessboard pattern were included. By placing either a purple or a blue color filter over the picture, two versions of each of the 50 pictures were created, resulting in 100 picture stimuli. An additional set of 18 mixed social and control pictures served as practice trials. A computer screen with a resolution of 1024 x 768 pixels and a ‘‘Logitech Attack 3” joystick were employed. Total administration of the AAT took approximately 10 minutes.

Participants were instructed to push the joystick upon presentation of pictures with a blue filter, while the joystick had to be pulled for purple pictures. Pictures disappeared when the joystick was pushed or pulled all the way in the correct direction. Appearance of the next picture was initiated by the participant by moving the joystick back to the central position and pulling the trigger button of the joystick. An unambiguous relation between movements and approach-avoidance behaviors was created by using a ‘zooming-effect’: When the joystick was pushed, the picture became smaller, and vice versa when it was pulled. This created the impression that the picture disappeared or came closer, respectively. The time from appearance to disappearance of the picture was automatically recorded. These times were the participants' reaction times, used to compute the dependent variables of the analyses described below. In a study concerning implicit avoidance of spiders, the internal consistency of the AAT was adequate for a reaction time task [52].

#### Stressful life events

Using an adapted version of the 21-item Life Events Questionnaire, which has good psychometric properties [53], participants were asked to indicate whether they had experienced a set of concrete life events before the age of 16 years, after the age of 16 and/or within the last year. In line with Vrijsen and colleagues [20,21], a childhood trauma variable was calculated including the following four interpersonal trauma items: physical/verbal aggression within the family, physical/verbal aggression outside the family, sexual abuse within the family and sexual abuse outside the family. This variable was coded ‘1’ when participants had experienced one of these events before the age of 16 years, and ‘0’ if they had not experienced these events before the age of 16.

#### Genotyping

A blood sample was taken via venipuncture. DNA was isolated using standard protocols. The 5-HTTLPR polymorphism was genotyped at the Department of Human Genetics of the Department of Human Genetics of the Radboud University Nijmegen Medical Center using Taqman analysis [54]. Two genotype groups were created based on allele frequency and in line with previous studies [55,56,57]: L_a_L_a_ (LL) versus S/Lg-carriers (SL_a_/L_a_L_g_ and SS/SL_g_/L_g_L_g_ combined)_._ The allele frequency distribution was L_a_L_a_: N = 54 (26%), SL_a_/L_a_L_g_: N = 109 (52%), SS/SL_g_/L_g_L_g_: N = 46 (22%). Testing for Hardy–Weinberg equilibrium (HWE), which was adjusted for multi-allelic variants, did not show deviations from the expected distribution of genotypes (*X*^2^ = .42, df = 1, *p* = .52).

#### Depression and other measures

All measures were administered before the AAT. Depressive symptomatology was measured with the Beck Depression Inventory-II (BDI-II) [58,59]. The BDI-II is a 21-item, self-report instrument that measures severity of depression, and has good to excellent psychometric properties [60]. Social anxiety was measured with the anxiety scale (24 items) of the Liebowitz Social Anxiety Scale [61]. The LSAS had good psychometric properties [62]. Age of onset of the first depressive episode, as well as the number of depressive episodes were assessed with separate questions and confirmed during the clinical interview (SCID-I). Current use of medication prescribed for anxiety, mood or sleep problems was assessed by a psychiatrist (author AS).

### Design and statistical analyses

To reduce the influence of outlying data points, RTs in the top and bottom 1% of the distribution were eliminated. Initial pull instead of push full movements and vice versa were considered errors. On average, error rates were low, less than 1%. Median RTs were computed for each participant for each combination of facial expression (angry, sad, happy, neutral) and movement (push, pull). An *approach-avoidance score* was calculated per facial expression by subtracting the median scores of pull trials from the push trials (push–pull). Negative approach-avoidance scores indicate faster avoidance than approach, and positive values indicate faster approach than avoidance. These approach-avoidance scores were used as the dependent variable in the analyses of the AAT. Differences between the two genotype groups on diverse demographic and clinical measures were analyzed with *t*-tests and Chi-square tests. Because of their known effect on cognitive functioning and emotional processing, age and sex were included as covariates in the main analyses. The 3-way interaction of the between-subjects factors Genotype (L_a_L_a_ vs. S/L_g_-carriers) and Childhood trauma (no vs. yes) with the within-subjects factor Facial expression (angry, sad, happy, neutral) on approach-avoidance scores was analyzed using repeated-measures ANCOVA.

## Results

Sample descriptives and statistical tests comparing the four combined childhood trauma (no, yes) and genotype (LaLa, S/Lg-carriers) groups on demographic and clinical variables are presented in Table 1. The groups did not differ on age, sex distribution, medication use, BDI-II total score, LSAS total score, age of depression onset, or number of past depressive episodes. Last, the genotype groups L_a_L_a_ (28%) and S/Lg-carriers (39%) did not differ in terms of percentage of experienced childhood trauma, *χ*2(1, N = 209) = 2.32, *p* = .128.

**Table 1:** Statistical tests comparing the combined childhood trauma (no, yes) and genotype (LaLa, S/Lg-carriers) groups on demographic and clinical variables. Means (Standard Deviations) or percentages are presented per variable.

	Group				
Variables	LaLa + No childhood trauma (N = 39)	LaLa + Childhood trauma (N = 15)	S/Lg-carriers + No childhood trauma (N = 94)	S/Lg-carriers + Childhood trauma (N = 61)	Statistical test
Age	50.5 (11.31)	45.67 (9.15)	49.57 (12.23)	48.64 (10.27)	*F*(3,205) = .75, *p* = .522
Sex, female	64%	73%	54%	72%	*χ*2(3, N = 209) = 5.94, *p* = .114
Medication use	51%	47%	47%	54%	*χ*2(3, N = 208) = .771, *p* = .856
BDI-II	15.4 (7.57)	14.23 (13.60)	14.14 (10.10)	15.31 (10.23)	*F*(3,205) = .24, *p* = .869
LSAS	23.8 (14.72)	18.68 (13.76)	18.82 (11.68)	20.67 (12.62)	*F*(3,205) = 1.51, *p* = .214
Age of onset	31.9 (12.31)	22.83 (10.39)	29.89 (12.64)	26.97 (13.36)	*F*(3,205) = 2.55, *p* = .057
Number of episodes	1.7 (.442)	1.93 (.258)	1.80 (.404)	1.84 (.373)	*F*(3,205) = .98, *p* = .405

Note. BDI-II refers to the score on the Beck Depression Inventory, LSAS refers to the Liebowitz Social Anxiety Scale, Age of onset refers to the age of the first depressive episode and Number of episodes refers to the number of depressive episodes (both indicated by the patient).

### 5-HTTLPR genotype and childhood trauma interaction on approach-avoidance tendencies

The 5-HTTLPR Genotype (L_a_L_a_ vs. S/Lg carriers) x Childhood trauma (no vs. yes) x Facial expression (angry, sad, happy, or neutral) ANCOVA yielded no main effect of 5-HTTLPR Genotype, nor main effects of Childhood trauma or Facial expression on approach-avoidance scores, *F*(1,203) = .05, *p* = .828, ηp2 < .001, *F*(1,203) = 2.74, *p* = .099, ηp2 = .013, and *F*(3,201) = .30, *p* = .827, ηp2 = .004, respectively.

As predicted, the ANCOVA did yield a significant three-way interaction of 5-HTTLPR Genotype x Childhood trauma x Facial expression on approach-avoidance scores, *F*(3,201) = 2.94, *p* = .034, η2 = .042. To rule out the possible role of current depressive and (social) anxiety symptoms, the Genotype x Childhood trauma x Facial expression ANCOVA was repeated in participants with a BDI-II Total score < 20 (N = 143), as well as in participants with an LSAS anxiety score < 19 (N = 104; based on the median LSAS score in our sample). Both analyses yielded similar results to the analysis in the full sample, *F*(3,135) = 4.56, *p* = .004, ηp2 = .09 for the BDI-II selection, and *F*(3,96) = 2.42, p = .070, ηp2 = .07 for the LSAS selection. Because the trauma and genotype groups differed at trend level on age of depression onset (see Table 1), and because age of onset is an important clinical predictor of depression severity and recurrence [63], its possible role was ruled out by including it as a covariate in the initial Genotype x Childhood trauma x Facial expression ANCOVA. This analysis also revealed very similar results compared with the initial ANCOVA, *F*(3,200) = 2.97, *p* = .033, ηp2 = .043.

The 5-HTTLPR Genotype x Childhood trauma interaction was significant for responses to sad facial expressions, *F*(1,203) = 6.73, *p* = .010, ηp2 = .032. Post-hoc power analyses using the G*power power calculator (http://www.gpower.hhu.de) showed that the power for the 5-HTTLPR x Childhood trauma interaction, at the 5% level, was 85%. For the facial expressions happy, angry and neutral, no significant two-way interaction was found, *F*(1,203) = 2.90, *p* = .09, ηp2 = .014, *F*(1,203) < .01, *p* = .964, ηp2 < .001, and *F*(1,203) = 1.70, *p* = .685, ηp2 = .001, respectively.

**Fig 1:**
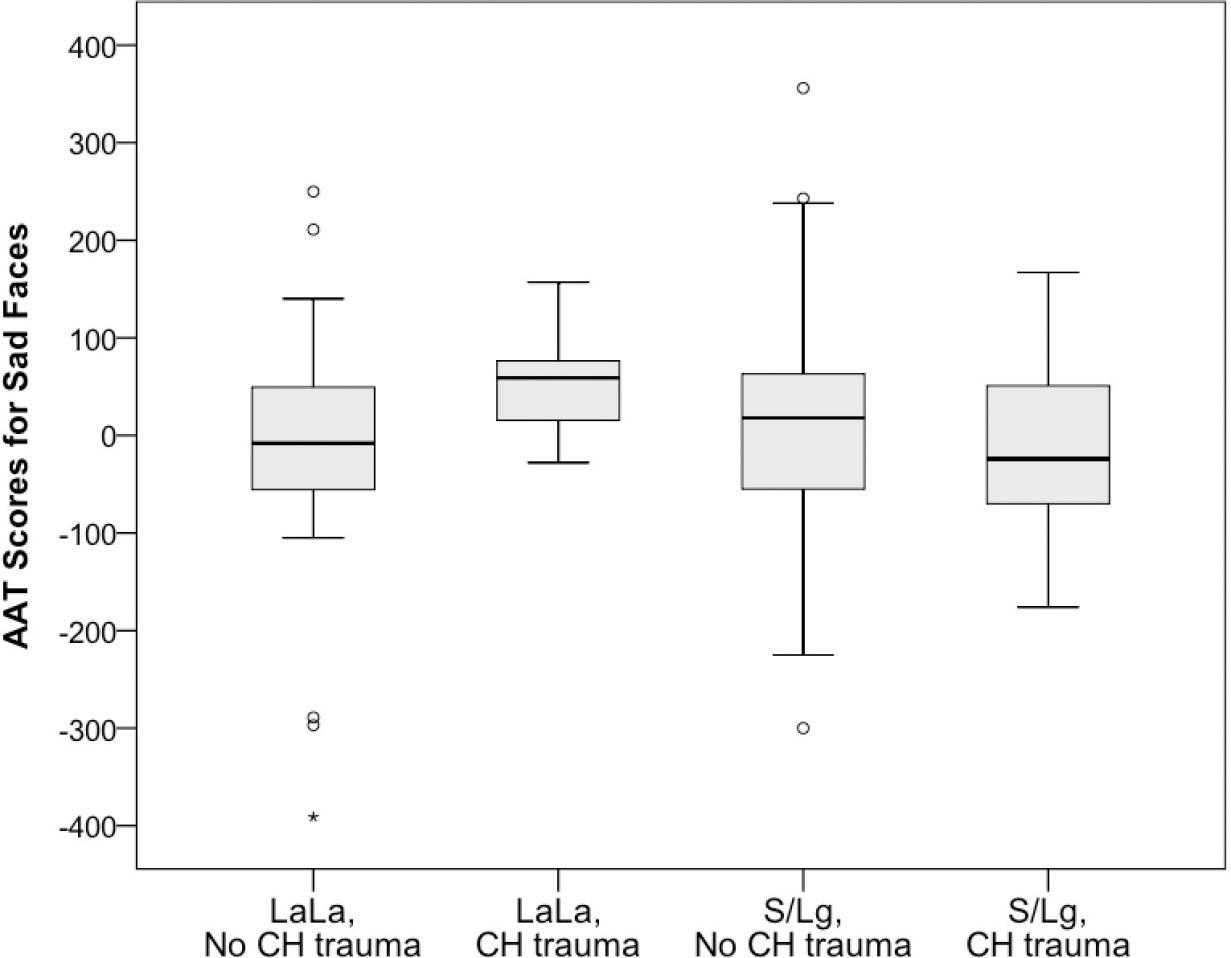
Median approach-avoidance scores (ms), interquartile range, outliers and extreme cases for sad faces, for the combined childhood trauma (yes vs. no) and genotype groups (S/Lg-carriers vs. LaLa).

Further post-hoc analyses of responses to sad faces revealed that remitted depressed S/L_g_-carriers with childhood trauma (*M* = -7, *SD* = 85) avoided sad faces relatively more compared to patients with the L_a_L_a_ genotype with childhood trauma (*M* = 57, *SD* = 54), *F*(1,72) = 9.05, *p* = .004, ηp2 = .112 (Fig 1). Furthermore, L_a_L_a_ individuals without childhood trauma (*M* = -12, *SD* = 122) avoided sad faces relatively more than L_a_L_a_ individuals with childhood trauma (*M* = 57, *SD* = 54), *F*(1,50) = 4.46, *p* = .040, partial ηp2 = .082. The two ANCOVA’s comparing the approach-avoidance scores for sad expressions of S/L_g_-carriers without childhood trauma (*M* = 14 , *SD* = 98) to L_a_L_a_ individuals with trauma, and the scores of S/L_g_-carriers without childhood trauma to S/Lg-carriers with childhood trauma did not reveal significant differences, for both *p* > .26.

### Comparison of genotype and childhood trauma groups on clinical variables

Depression characteristics are associated with depression severity, persistence and level of adjustment after a recovery from a depressive episode [64]. Therefore, post-hoc analyses were used to study possible differences in the age of onset of the first depressive episode and number of depressive episodes, which might account for the results found on approach-avoidance tendencies towards sad faces. However, the 5-HTTLPR Genotype x Childhood trauma interaction was not significant, neither for age of onset of the first depressive episode nor for number of depressive episodes, *F*(1,203) = 1.54, *p* = .217, ηp2 = .008, and *F*(1,203) = 1.57, *p* = .212, ηp2 = .008, respectively.

## Discussion

Within a naturalistic sample of remitted depressed patients, we found preliminary evidence for differential automatic avoidance of sad facial expressions based on interindividual differences in 5-HTTLPR genotype and the experience of childhood trauma. S/L_g_-carrying remitted depressed patients with childhood trauma automatically avoided sad facial expressions relatively more than L_a_L_a_ homozygotes with childhood trauma, even after controlling for age of depression onset, residual depressive symptoms, and social anxiety. Although tentative, this result fits with previous results suggesting that S/Lg-carriers show more avoidance behavior than L_a_L_a_-carriers [26,28], and that they may be most susceptible to the effects of childhood trauma [11]. It also conforms with findings that S/Lg-carriers generally show less emotional and psychological resilience than L_a_L_a_-carriers [65]. Variance in approach-avoidance tendencies indicates differential automatic response to emotional material [38]. Together, this preliminary result may indicate that automatic approach-avoidance tendencies are not only a symptom of depression, but may be a marker for depression vulnerability.

Secondly, and contrary to our expectation that pronounced differences in biased approach-avoidance tendencies would be found in the more vulnerable S/Lg-carriers, the present preliminary results showed that remitted depressed patients with the L_a_L_a_ genotype who had *not* experienced trauma during childhood avoided sad faces relatively more than L_a_L_a_-homozygotes with childhood trauma. This is unexpected for several reasons. As stated above, L_a_L_a_-carriers are usually the least vulnerable group. Furthermore, the L_a_L_a_ genotype is generally not associated with avoidance and depressotypic behavior, nor with susceptibility to environmental factors such as childhood trauma [66]. Last, from a clinical perspective, one might expect avoidance in people with childhood trauma, and not in those who did not experience it. Although speculative until replicated, this result may indicate that the generally less vulnerable L_a_L_a_-homozygotes who experience adversity during childhood are perhaps best equipped to recover from trauma and even face-related cues.

In line with Seidel and colleagues [42], we expected automatic avoidance of angry faces as well. We did not find this, however. The present results are tentative, but perhaps the signaling of sad rather than angry expressions is most relevant for depression. This would correspond with information processing models in depression: Sad faces are congruent to depressed patients’ mood state, activating depressotypic schemas, which lead to biased processing. This is corroborated by our finding that symptoms of social anxiety (LSAS) did not influence results.

Surprisingly, and also in contrast to our expectations and findings emphasizing the role of childhood trauma in depression [8,9], we did not find a main effect of childhood trauma, nor did S/L_g_-carriers with childhood trauma show more automatic avoidance of sad expressions than S/Lg-carriers without childhood trauma. Due to the tentative nature of this study, we cannot draw firm conclusions about this finding. Although tentatively, it might show, however, that childhood trauma does not exclusively contribute to depression. Rather, its effect on depressive behavior may depend on underlying biological variation, such as variance in the 5-HTTLPR polymorphism.

Taken together, the present findings correspond with previous studies showing an association between 5-HTTLPR and avoidance [26,28], and models of biased information processing in depression [67,68]. In line with other intermediate phenotype findings, our conceptual replication partly supports earlier G x E findings, linking automatic biased processing of emotional information to genetic risk profiles and childhood trauma [10,11,14,16–22]. We extended these findings to a naturalistic sample of (previously) depressed individuals and specifically found that automatic avoidance tendencies of sad faces might be one of the intermediate phenotypes mediating the relationship between genes and depression vulnerability. However, the specific pattern of results was (partly) unexpected.

These results are preliminary and replications are needed before firm conclusions can be drawn, so possible clinical considerations are speculative. If substantiated in larger samples, the results might contribute to the development of more personalized healthcare, targeting patients’ specific risk factors such as genetic and cognitive profiles [69,70]. In line with Acceptance and Commitment Therapy [71], the present study stresses the possibility of experiential avoidance of negative emotions and cognitions as a crucial factor in (the recurrence of) depression, and possible working mechanisms for intervention. The degree to which automatic avoidance remains present after treatment might be an independent predictor of recurrence of depression. In line with Cognitive Bias Modification (CBM) interventions, such as the approach-positivity training for depression developed by Becker and colleagues [72], it would be interesting to study the feasibility of directly modifying automatic approach-avoidance tendencies in depression [73]. The 5-HTTLPR gene allelic variations might alter sensitivity to CBM [74], underscoring the relevance of identifying genetic profiles in depressed patients. Possible future clinical applications such as these are still hypothetical and, of course, more research is needed.

The present findings cannot be interpreted without discussing several limitations of the study. The relatively modest sample size of the present study (N = 209) must be taken into account when evaluating the results. Post-hoc power analyses [75], however, showed that power was sufficient for detecting the 5-HTTLPR x Childhood trauma interaction. Although we consider our naturalistic sample of remitted depressed patients as typical and representative, studying such a group has disadvantages. Biased processing, for example, might reflect a susceptibility factor as well as scars from previous depressive episodes [76]. Relatedly, this is neither a prospective nor an experimental study, so no causal interpretations can be drawn. Another limitation is the method of assessing childhood trauma with the self-report Life Events Questionnaire [53]. Although others state that adults' recall of specific childhood events seems fairly accurate [77], relying on self-report could have confounded the present results to some extent [10]. In line with Vrijsen and colleagues [22], we used a rather stringent selection of childhood trauma events. This might have resulted in a lower incidence of childhood trauma than in related studies [78].

However, despite these limitations, the tentative results of this explorative study suggest that automatic approach-avoidance tendencies might be a candidate intermediate phenotype for depression. The specific pattern of these tendencies may depend on genetic profile and the experience of childhood trauma. Although this might endorse the complex and multilateral etiology of depression, and the relevance of integrating depression risk factors at different levels (genetic, information processing, psychosocial), replications using larger samples are needed for substantiation of the findings and for clarifying the specific association between genetic variance, childhood experiences and approach-avoidance behavior.
